# Inference about time-dependent prognostic accuracy measures in the presence of competing risks

**DOI:** 10.1186/s12874-020-01100-0

**Published:** 2020-08-28

**Authors:** Rajib Dey, Giada Sebastiani, Paramita Saha-Chaudhuri

**Affiliations:** 1 Department of Epidemiology, Biostatistics and Occupational Health, McGill University, Montreal, Canada; 2 Division of Gastroenterology and Hepatology, McGill University Health Centre, Montreal, Canada; 3 Department of Mathematics and Statistics, University of Vermont, Burlington, USA

**Keywords:** Competing Risks, Area under the ROC curve (AUC), Cause-specific AUC, Fractional Polynomials

## Abstract

**Background:**

Evaluating a candidate marker or developing a model for predicting risk of future conditions is one of the major goals in medicine. However, model development and assessment for a time-to-event outcome may be complicated in the presence of competing risks. In this manuscript, we propose a local and a global estimators of cause-specific AUC for right-censored survival times in the presence of competing risks.

**Methods:**

The local estimator - cause-specific weighted mean rank (cWMR) - is a local average of time-specific observed cause-specific AUCs within a neighborhood of given time *t*. The global estimator - cause-specific fractional polynomials (cFPL) - is based on modelling the cause-specific AUC as a function of *t* through fractional polynomials.

**Results:**

We investigated the performance of the proposed cWMR and cFPL estimators through simulation studies and real-life data analysis. The estimators perform well in small samples, have minimal bias and appropriate coverage.

**Conclusions:**

The local estimator cWMR and the global estimator cFPL will provide computationally efficient options for assessing the prognostic accuracy of markers for time-to-event outcome in the presence of competing risks in many practical settings.

## Background

In modern evidence-based medicine, decisions on appropriate early medical intervention or the choice and timing of interventions frequently rely on prognostic markers that are informative for survival outcome. Such marker can be a single covariate or a risk score where the latter is estimated from a survival model of the association between covariates and survival time. For instance, a well-known Framingham risk score predicts the 10-year risk of cardiovascular disease [1]. This model often guides clinicians to identify those at high/low-risk for future cardiovascular disease. Similar risk models and markers exist for other diseases, e.g. Gail score for breast cancer [2], prostate specific antigen for prostate cancer, kidney donor risk index (KDRI) of donor for kidney transplant etc. In liver transplantation study (LT), the side-effects of medications put the organ recipients at risk for recurrent liver disease (liver fibrosis) which can lead to graft failure [3]. Liver biopsy is the gold standard for staging, managing and treating liver fibrosis, and useful in the prognosis of LT recipients. However, biopsy is often impractical due to its invasiveness and higher cost. Serum fibrosis markers such as the fibrosis score 4 (FIB-4), and the nonalcoholic fatty liver disease fibrosis score (NAFLD) measured one year after LT have recently been shown to accurately stratify organ recipients who are at high risk from those who are at low risk of recurrent liver disease [3], [4]. Therefore, rather than performing biopsy on all patients, these markers could guide clinicians to start timely treatment for those high-risk patients while sparing low-risk patients from the side effects of biopsy and from the unnecessary costs. Before one adopts such markers into clinical practice, it is crucial to evaluate their prognostic accuracy i.e. whether markers correctly discriminate subjects who will subsequently experience the event of interest at or by time *t* from those who will not experience any event by *t*. The goal of this paper is to develop a method to estimate time-dependent prognostic accuracy measure of such marker.

The evaluation of prognostic accuracy of marker becomes complicated in the presence of competing risks. Competing risks arise when a subject experiences a terminal event due to one of the multiple mutually exclusive causes. For example, post-liver transplant, patients may die due to adverse liver and/or transplant-related outcomes (e.g. graft failure) while other patients may die due to competing events (e.g. non-liver causes) before experiencing liver-related adverse outcomes. This leads to a competing risks situation because liver-related marker could predict graft failure or graft related death, but may not predict non-graft related events. [5]. Here, the scientific question is how well do FIB-4 or NAFLD discriminate between patients who progress to graft-related death and those who do not. Understanding whether FIB-4 or NAFLD is highly predictive for death due to graft failure but not others could potentially lead to more rational and cost-effective use of specific medications or treatment strategies. In order to facilitate the assessment of prognostic accuracy of marker, the goal of this manuscript is to develop methods to estimate time-dependent prognostic accuracy of a baseline marker after taking right-censoring and competing risks into account.

A number of statistical measures have been proposed to assess the prognostic accuracy of a marker. The Receiver operating characteristic (ROC) curve and the area under the ROC curve (AUC) [6] are the most popular measures of a binary classifier system. ROC curve is a graphical illustration of the diagnostic ability of a binary classifier system as its discrimination threshold is varied and AUC provides a global summary of the discriminatory capacity of the marker. For a survival outcome, the event status of a subject can change over time and the risk of developing the event conditional on marker value changes over the follow-up time. Therefore, the accuracy summaries for evaluating the performance of a marker must take this time-dependence into account. Time-dependent ROC methods for survival time [7],[8] classify the subjects as cases or controls depending on their survival status at or by time *t* and compare their observed status with a predicted risk at some or all times. The incident cases/dynamic controls classification arises [8] when scientific interest focuses on correct classification of subjects who are still in risk at time *t*. The incident (I) cases are those subjects who had an event at *t* and the dynamic (D) controls are those who survived through *t*. Among the other definitions of time-dependent ROC, cumulative (C) case and dynamic control definition pair is most common. In this classification, cumulative cases are defined as patients having an event within a certain time range, say [0,*t*]. The I/D version of AUC focuses on incident cases and is better suited for characterizing the trajectory of AUC over time, while C/D AUC does not characterize the evolution of accuracy over time. Another appealing property of I/D measure is that the evaluation of the model at a certain time point *t* only focuses of the riskset at the time *t* and therefore prior events and performance of the marker does not influence or distort the marker accuracy at *t*. As prognostic models aim to predict future, this property is very appealing when evaluating dynamic prognostic models.

In this article, we focus on I/D definition of AUC(*t*), which is the time-dependent area under the I/D ROC curve at *t* and was introduced in [8]. There has been extensive research in estimating the I/D AUC(*t*) in the case of single cause of failure. In [9], a locally weighted mean rank (WMR) smoothing based on the intermediate concordance measure was proposed. A fractional polynomials (FPL) estimator based on modeling AUC(*t*) as a function of time was proposed in [10]. To evaluate the methods, the authors in [11] compared different approaches (i.e. [8], [12], [6]) of estimating the concordance index based on the AUC(*t*) by simulation studies. However, the estimation of the AUC(*t*) at different follow-up time points was not investigated in that study.

Though competing risks is an important issue in many practical clinical settings, there is limited research on estimating the I/D AUC(*t*) in the presence of competing risks. When there is only a single cause of failure, the I/D definition stratifies only the subjects in the riskset. In the presence of competing risks, I/D definition further stratifies the subjects who are still at risk at time *t* into a single control group and cause-specific case groups depending on the cause of failure. Estimation of time-dependent measures under competing risks were discussed in [13] and [14]. In [13] a time-dependent I/D ROC curve was estimated using a Cox model for the cause-specific hazards and riskset reweighting of the marker distribution. This approach is semi-parametric, indirect, and computationally intensive. First, it requires correct specification of a conditional hazard regression model linking the marker to the event time. This approach provides biased estimate when the monotonicity of association between marker and event time is violated. Second, in order to obtain the AUC curve from the ROC, numerical integration of the ROC curve is required. Furthermore, within an interval around each unique event time, a Cox model is assumed and the parameters of the sequence of Cox models are estimated for each neighborhood around the unique event times. Therefore, their approach is also time-consuming. The goal of this manuscript is to propose non-parametric, direct, intuitive and scalable methods to estimate the I/D cause-specific AUC(*t*) of baseline marker, accounting for censoring and competing events. The major advantage of our proposed method over the existing semi-parametric estimator will be that it requires no specification of a conditional hazard model linking the marker to the event time and hence robust to model misspecification. In addition, the inference of this measure can be developed under minimal assumptions.

The rest of the article is organized as follows. In “Notation” section, we provide the notation. In “Weighted mean rank estimator of i/D cause-Specific aUC(*t*) (cWMR)” and “Fractional polynomial estimator of i/D cause-Specific aUC(*t*) (cFPL)” sections, we introduce a local and a global estimators of cause-specific I/D AUC(*t*) that are direct, flexible and non-parametric. We report simulation results to illustrate our methodology in “Simulation study” section. We report a real data example in ‘Application” section, and put a discussion in “Discussion” section. We end with conclusion in “Conclusions” section.

## Methods

### Notation

Let *M* denote the (baseline) marker that could potentially be used in predicting the survival time in the presence of competing risks where a subject can fail due to *J* mutually exclusive causes. Note that *M* can be a single covariate or it could be a risk score that may be calculated from a survival model (e.g. proportional hazard model). Let the implicit event times for each of the *J* causes be $\{{T}^{(1)},\dots,{T}^{(J)}\}$. In the presence of competing risks, only the time to the occurrence of the first event is potentially observable. Thus, without censoring, one observes *T* = min $({T}^{(1)},\dots,{T}^{(J)})$ and the failure indicator *δ*, which takes value *j* if $T={T}^{(j)} (j=1,\dots J)$. Define the observed event time, *Z* = min (*T*,*C*) where *C* is the censoring time. A censored observation has *Z*=*C* and this is recorded by *δ*=0. Suppose that there are *n* subjects in the study. Let **1**(.) denote the indicator function. Let *R*_*i*_(*t*)=**1**{*Z*_*i*_≥*t*} denote the at-risk indicator for the *i*-th individual at time *t* (*i*=1,2,...,*n*). Let $\mathbb {R}_{t}=\{i:R_{i}(t)=1\}$ denote the subjects that are in the riskset at time *t*. Among the subjects in $\mathbb {R}_{t}$, the subjects who had an event from cause *j* at *t* are the *j*-th cause-specific incident (I) cases: $\mathbb {R}^{(j)}_{1t}=\{{i:T_{i}=t,\delta _{i}=j}\}$. The subjects who did not have an event by *t* are the dynamic (D) controls: $\mathbb {R}_{0t}=\{i:T_{i}>t\}$. Let *n*_*t*_ be the size of $\mathbb {R}_{0t}$ i.e. $n_{t}=|\mathbb {R}_{0t}|$ and $d^{(j)}_{t}$ be the size of $\mathbb {R}^{(j)}_{1t}$, $d^{(j)}_{t}=|\mathbb {R}^{(j)}_{1t}|$.

If there is a single cause of failure (*J*=1), the time-dependent incident/dynamic area under the ROC curve at time *t*, I/D AUC(*t*), is defined as

1 $$ \text{Pr}(M_{i}>M_{k}|T_{i}=t,T_{k}>t).  $$

This is the time-dependent probability at time *t* that for a randomly selected case-control pair (*i*,*k*) the marker value for the incident case is higher than the marker value for the control.

### Time-dependent i/D cause-Specific aUC(*t*)

In the presence of competing risks, the I case and D control definition can be extended as cause-specific incident cases and dynamic controls as follows:

- *j*-th cause-specific case: $T = t,\delta =j;\,\,\, j=1,2,\dots,J.$
- Control: *T*>*t*.

The I/D AUC(*t*) in (1) can be redefined as the *j*-th I/D cause-specific AUC(*t*) and define as

2 $$ \text{AUC}^{(j)}_{t}=\text{Pr}(M_{i}>M_{k}\mid T_{i}=t,\delta_{i}=j, T_{k}>t),  $$

where $j=1,2,\dots,J.$ Below we propose a local and a global estimators for the time-dependent cause-specific AUC(*t*) curve.

### Weighted mean rank estimator of i/D cause-Specific aUC(*t*) (cWMR)

A non-parametric estimator of I/D AUC(*t*) was proposed in [9] using a nearest neighbor method. This estimator is a local average of time-specific observed AUCs. While the method do not address the issue of more than one cause of failure, natural modification of the approach allows estimation of accuracy in the presence of multiple causes of failure. To illustrate our proposed method, let A^(*j*)^(*t*) denote the proportion of (*i*,*k*) pairs where subject *k* has a lower marker value compared with that of subject *i* who experienced failure due to cause *j*, provided subject *k* has longer survival than subject *i* and define as

$${\begin{aligned} \mathrm{A}^{(j)}(t)=\frac{1}{d^{(j)}_{t}\times n_{t}} \sum_{i\in \mathbb{R}^{(j)}_{1t}} \sum_{k\in \mathbb{R}^{0}_{t}} \textbf{1}\{M_{i}>M_{k}\mid T_{i}=t,\delta_{i}=j, T_{k}>t\}. \end{aligned}} $$

Note that, A^(*j*)^(*t*) can be considered as an estimator of $\text {AUC}^{(j)}_{t}$ in (2). However, typically failure time is measured in continuous scale and it is reasonable to assume that at a given time the likelihood of failures due to multiple causes is negligible. Therefore, there are only few cases experiencing the event of interest (e.g. *j*-th cause) at *t* and often $d^{(j)}_{t}= 1$. When $d^{(j)}_{t} = 1$, $ \mathrm {A}^{(j)}(t)=\frac {1}{n_{t}} \sum _{k\in \mathbb {R}^{0}_{t}} \textbf {1}\{M_{i}>M_{k}\mid T_{i}=t,\delta _{i}=j, T_{k}>t\} $ is the rank of the *j*-th cause-specific case marker value among the control markers at time *t*. In extreme situations, this quantity could jump from 1 to 0 and back between adjacent time points. Hence, the estimation of $\text {AUC}^{(j)}_{t}$ based on A^(*j*)^(*t*) requires some degree of smoothing. In this situation, the information within a neighborhood around *t*, ${N}^{(j)}_{t}(h_{n})=\{t_{k}: |t-t_{k}|< h_{n},\delta =j\}$ can be used to estimate marker concordance at *t*. Let $\text {cWMR}^{(j)}_{t}$ be an *j*-th cause-specific weighted mean rank estimator of $\text {AUC}^{(j)}_{t}$ and define as

3 $$ \text{cWMR}^{(j)}_{t}=\frac{1}{|N^{(j)}_{t}(h_{n})|} \sum_{t_{k}\in N^{(j)}_{t}(h_{n})} \mathrm{A}^{(j)}(t_{k}), \vspace{-2mm}  $$

where $|N^{(j)}_{t}(h_{n})|$ is the size of $N^{(j)}_{t}(h_{n}).$ The optimal bandwidth (*h*_*n*_) balances bias and variance of $\text {cWMR}^{(j)}_{t}$. Therefore, to select the bandwidth, we followed the leave-one-out cross validation approach of [9] and adapted it to account for competing risks. In addition, we also propose a variance estimator for $\text {cWMR}^{(j)}_{t}$ based on the assumption of bivariate Normality of the case and control marker pairs [9]. An additional file shows this in more detail [see Additional file 1].

#### Asymptotic properties of $\,\,\text {cWMR}^{(j)}_{t}$:

In order to evaluate the asymptotic behaviours of $\,\,\text {cWMR}^{(j)}_{t},$ we restrict our attention to a neighborhood around *t* and cause *j* i.e. ${N}^{(j)}_{t}(h_{n})=\{t_{k}: |t-t_{k}|< h_{n},\delta =j\}$ with size $|N^{(j)}_{t}(h_{n})|=m^{(j)}_{t}$. Other causes can be treated in a similar way. Let *B*_*t*_ denote the number of subjects at the start of the neighborhood i.e. at time *t*−*h*_*n*_.

##### **Theorem 1**

Let $n-\left (B_{t}+m_{t}^{(j)}\right)$ denote the number of subjects surviving past *t*+*h*_*n*_. If $n-\left (B_{t}+m_{t}^{(j)}\right)\rightarrow \infty $ as *n*→*∞* and *h*_*n*_→0 then $\text {cWMR}^{(j)}_{t}$ converges to a Normal distribution with mean $\text {AUC}^{(j)}_{t}+b_{n}(t)$ and asymptotic variance ${\mathrm {V}^{(j)}_{n}=\text {Var}[\text {cWMR}^{(j)}_{t}].}$ Here, *b*_*n*_(*t*) denotes the bias and the variance is

$${\begin{aligned} { \mathrm{V}^{(j)}_{n}=\frac{1}{[m^{(j)}_{t}]^{2}}\{\sum_{t_{(i)}\in N^{(j)}_{t}(h_{n})} \text{var}[\mathrm{A}^{(j)}(t_{(i)})]+\sum_{t_{(i)}\neq t_{(k)}}\text{cov}[\mathrm{A}^{(j)}(t_{(i)}),\mathrm{A}^{(j)}(t_{(k)})]\}.} \end{aligned}} $$

The proof of this theorem is provided in Additional file 1.

#### Estimation of variance of $\text {cWMR}^{(j)}_{t}$

The variance of $\text {cWMR}^{(j)}_{t},\mathrm {V}^{(j)}_{n}$, has components var[A^(*j*)^(*t*_(*i*)_)] and cov[A^(*j*)^(*t*_(*i*)_),A^(*j*)^(*t*_(*k*)_)]. In order to compute these components, we propose the following estimators in the spirit of variance calculation of AUC proposed in [15] and [9]

$${\begin{aligned} {\text{var}[\mathrm{A}^{(j)}(t)]=(\frac{ n_{t}-1}{n_{t}})\{P_{1}^{(j)}(t)-[P_{0}^{(j)}(t)]^{2}\}+\frac{1}{n_{t}}\{P_{0}^{(j)}(t)(1-P_{0}^{(j)}(t))\}} \end{aligned}} $$

$${\begin{aligned} {\text{cov}[\mathrm{A}^{(j)}(t),\mathrm{A}^{(j)}(s)]=\frac{1}{n_{t}}[\{P_{2}^{(j)}(t,s)-P_{2.0}^{(j)}(t,s)\}+\{P_{3}^{(j)}(t,s)-P_{3.0}^{(j)}(t,s)\}].} \end{aligned}} $$

We estimate $P_{0}^{(j)}(.),P_{1}^{(j)}(.)$ etc. using a Normal approximation for the *j*-th cause-specific case and control markers after a rank-based Z-score transformation and then empirically estimating the parameters of the approximating normal distributions. The detail is provided in Additional file 1.

### Fractional polynomial estimator of i/D cause-Specific aUC(*t*) (cFPL)

The authors in [10] proposed a method for modelling I/D AUC(*t*) defined in equation (1) in the case of single event type. Their method directly models AUC(*t*) as a function of the event time *t* through a flexible fractional polynomials model proposed in [16]. We have extended it in the presence of competing risks as follows.

The $\text {AUC}^{(j)}_{t}$ after transformation with link function *η* can be specified as a parametric function of *t* using fractional polynomials of degree *L*:

4 $$ \eta\left(\text{AUC}^{(j)}_{t}(\beta_{j})\right)=\beta_{j0}+\sum_{l=1}^{L} \beta_{jl}t^{(p_{l})},  $$

where for *l*=1,2,...,*L*

$$t^{(p_{l})}=\left\{ \begin{array}{ccc} t^{p_{l}} &\ \text{if} &\ p_{l} \neq 0\\ \text{ln}(t) & \text{if} &\ p_{l}= 0, \end{array}\right. $$

with $p_{1}\leq p_{2}\leq \dots \leq p_{L}$ real-valued powers. As suggested in [10], we consider the power set $p_{1},\dots,p_{L}$ in {-2,-1,-0.5,0,0.5,1,2}, which is flexible enough to accommodate most applications. The set of regression parameters $\beta _{j}=(\beta _{j0},\beta _{j1},\dots,\beta _{j7})$ is then estimated by optimizing a likelihood function. A logit function considered for *η*(.) similar to [10]. Let there are *h*_*j*_ number of failures due to cause *j* in the study. For each event time {*t*_(*H*)_ & *δ*_*H*_=*j*}, there are two types of random variables

$${}n_{1}(t_{(H)})\,=\,\!\sum_{k} \textbf{\!1}\{k:M_{H}>\!M_{k}|Z_{H}\!=t_{(H)},\delta_{H}\!=j,k\in \mathbb{R}_{0t_{(H)}}\}, $$

and

$${}n_{2}(t_{(H)})\,=\,\sum_{k} \!\textbf{1}\{k:M_{H}\leq\! M_{k}|Z_{H}\!=t_{(H)},\delta_{H}\!=j,k\in \mathbb{R}_{0t_{(H)}}\}, $$

where *n*_1_(*t*_(*H*)_) and *n*_2_(*t*_(*H*)_) are the number of concordant and number of discordant pairs, respectively. Note that, conditional on riskset $\mathbb {R}_{0t_{(H)}}$, the count *n*_1_(*t*_(*H*)_) follows a Binomial distribution with probability $\text {AUC}^{(j)}_{t_{(H)}}(\beta _{j})$. The partial-likelihood for *j*-th cause of failure is

5 $$ {}L(\beta_{j}) = \prod_{H=1}^{h_{j}} \{\text{AUC}^{(j)}_{t_{(H)}}(\beta_{j})\}^{n_{1}(t_{(H)})} \{1-\text{AUC}^{(j)}_{t_{(H)}}(\beta_{j})\}^{n_{2}(t_{(H)})} \vspace{-2mm}  $$

Maximizing this partial-likelihood yields the parameter estimate $\hat \beta _{j}$ of *β*_*j*_. Then, by using (4), we obtain $\text {AUC}^{(j)}_{t}(\hat {\beta }_{j}$) estimate as a smooth function of time *t* and $\hat {\beta }_{j}$. For estimation, we evaluate the score equations that correspond to the proposed likelihood. The proposed likelihood is constructed in the spirit of the [10]. However, the main difference is that, unlike [10], we have multiple causes of failure. We can write the log-likelihood in the following way

$${{}\begin{aligned} l(\beta_{j})&\,=\,\!\sum_{i=1}^{n}\!\sum_{k=1}^{n}\!\delta_{ij}\,\,\textbf{1}\{Z_{k}>Z_{i}\}\,\,\{\textbf{1}\{M_{i}>M_{k}\}\,\,\text{log}(\text{AUC}^{(j)}_{Z_{i}}(\beta_{j}))\\&+\textbf{1}\{M_{i}\leqslant M_{k}\}\,\,\text{log}(1-\text{AUC}^{(j)}_{Z_{i}}(\beta_{j}))\}, \end{aligned}} $$

where for $j=1,2,\dots,J$

6 $$ \delta_{ij}=\left\{ \begin{array}{cc} 1, &\ \delta_{i}= j\\ 0, & \text{otherwise.} \end{array}\right.  $$

We estimate $\hat \beta _{j}$ of *β*_*j*_ as a solution of score vector *U*(*β*_*j*_)=0. The elements of *U*(*β*_*j*_) are

$${}\begin{aligned} \frac{d}{d\beta_{j0}}l(\beta_{j})&=\sum_{i=1}^{n}\sum_{k=1}^{n}\delta_{ij}\textbf{1}(Z_{k}>Z_{i})\{\textbf{1}\{M_{i}>M_{k}\}\\&\frac{\frac{d}{d\beta_{j0}}\text{AUC}^{(j)}_{Z_{i}}(\beta_{j})}{\text{AUC}^{(j)}_{Z_{i}}(\beta_{j})} -\textbf{1}\{M_{i}\leqslant M_{k}\}\frac{\frac{d}{d\beta_{j0}}\text{AUC}^{(j)}_{Z_{i}}(\beta_{j})}{1-\text{AUC}^{(j)}_{Z_{i}}(\beta_{j})}\}, \end{aligned} $$

where

$$\frac{d}{d\beta_{j0}}\text{AUC}^{(j)}_{Z_{i}}(\beta_{j})= \text{AUC}^{(j)}_{Z_{i}}(\beta_{j}) \{1-\text{AUC}^{(j)}_{Z_{i}}(\beta_{j})\}.$$

Again, for *l*=1,2,..,7, we get

$${}\begin{aligned} \frac{d}{d\beta_{jl}}l(\beta_{j})&=\sum_{i=1}^{n}\sum_{k=1}^{n}\delta_{ij}\textbf{1}\{Z_{k}>Z_{i}\}\{\textbf{1}\{M_{i}>M_{k}\}\\&\frac{\frac{d}{d\beta_{jl}}\text{AUC}^{(j)}_{Z_{i}}(\beta_{j})}{\text{AUC}^{(j)}_{Z_{i}}(\beta_{j})}-\textbf{1}\{M_{i}\leqslant M_{k}\}\frac{\frac{d}{d\beta_{jl}}\text{AUC}^{(j)}_{Z_{i}}(\beta_{j})}{1-\text{AUC}^{(j)}_{Z_{i}}(\beta_{j})}\}, \end{aligned} $$

where

$$\frac{d}{d\beta_{jl}}\text{AUC}^{(j)}_{Z_{i}}(\beta_{j})=Z_{i}^{(p_{l})} \times \text{AUC}^{(j)}_{Z_{i}}(\beta_{j}) \{1-\text{AUC}^{(j)}_{Z_{i}}(\beta_{j})\}. $$

In order to make inferences about the proposed estimators of the cause-specific parameters, the major challenge lies in the fact that the proposed partial-likelihood cannot be treated as a regular likelihood function. Specifically, the asymptotic variance of the estimators is not the inverse of the negative second derivative of the partial-likelihood. We propose a sandwich variance estimator for the proposed global cause-specific AUC(*t*) estimator below.

#### Asymptotic properties of cFPL estimator

In this section, we describe the asymptotic properties of the model parameter estimators. We state some regularity conditions in Additional file 1. We summarize the asymptotic behavior of the regression parameter estimator in the following theorem.

##### **Theorem 2**

Under the regularity conditions, $\hat {\beta _{j}}$ converges almost surely to *β*_0*j*_, while $\sqrt {n}(\hat {\beta }_{j}-\beta _{0j})$ converges to a multivariate Normal distribution with mean vector **0** and covariance matrix $\Sigma _{j1}^{-1}\Sigma _{j2}\Sigma _{j1}^{-1}$. Here,

$$ \Sigma_{j1}=\mathrm{E}\{-\frac{d}{d\beta_{j}}f_{ik}(\beta_{j});\beta_{0j}\}, $$

$$ \Sigma_{j2}=4\,\,\text{Cov}\{g_{ik}(\beta_{j}),g_{ik^{'}}(\beta_{j});\beta_{0j}\}, $$

where, $g_{ik}(\beta _{j})=\frac {(f_{ik}(\beta _{j})+f_{ki}(\beta _{j}))}{2}$ and

$$\begin{aligned} f_{ik}(\beta_{j})&=\int_{0}^{\tau} \int_{0}^{\tau} \textbf{1}\{t>s\}\{\textbf{1}\{M_{i}>M_{k}\}\frac{\frac{d}{d\beta_{j}}\text{AUC}^{(j)}_{Z_{i}}(\beta_{j})}{\text{AUC}^{(j)}_{Z_{i}}(\beta_{j})}\\&-\textbf{1}\{M_{i}\leqslant M_{k}\}\frac{\frac{d}{d\beta_{j}}\text{AUC}^{(j)}_{Z_{i}}(\beta_{j})}{1-\text{AUC}^{(j)}_{Z_{i}}(\beta_{j})}\}dN_{i}^{(j)}(s) dN_{k}^{(j)}(t), \end{aligned} $$

where $N_{i}^{(j)}(\tau)$ counts number of events due to *j*-th cause of failure occurring over [0,*τ*]. Theorem 2 can be proven in the spirit of the proof in [10]. However, the main difference is that, unlike [10], our likelihood construction account multiple causes of failure. An additional file shows the proof in more detail [see Additional file 1]. The covariance can be consistently estimated by $\hat {\Sigma }_{j1}^{-1}\hat {\Sigma }_{j2}\hat {\Sigma }_{j1}^{-1}$, where

$$\begin{aligned} \hat{\Sigma}_{j1}&=\frac{-1}{n^{2}}\sum_{i=1}^{n}\sum_{k=1}^{n}\delta_{ij}\,\textbf{1}\{Z_{k}>Z_{i}\}\,\, \frac{d}{d\beta_{j}}\{\textbf{1}\{M_{i}>M_{k}\}\\&\frac{\frac{d}{d\beta_{j}}\text{AUC}^{(j)}_{Z_{i}}(\hat{\beta}_{j})}{\text{AUC}^{(j)}_{Z_{i}}(\hat{\beta}_{j})}-\textbf{1}\{M_{i}\leqslant M_{k}\}\frac{\frac{d}{d\beta_{j}}\text{AUC}^{(j)}_{Z_{i}}(\hat{\beta}_{j})}{1-\text{AUC}^{(j)}_{Z_{i}}(\hat{\beta}_{j})}\}, \end{aligned} $$

and

$$\hat{\Sigma}_{j2}=\frac{4}{n(n-1)(n-2)}\sum_{i=1}^{n}\sum_{k\neq k^{'},k,k^{'}\neq i}g_{ik}(\hat{\beta}_{j})\, {g_{ik^{'}}(\hat{\beta}_{j})}^{T}. $$

**Corollary 1:** Under the regularity conditions, $\text {AUC}_{t}^{(j)}(\hat {\beta }_{j})$ is a consistent estimator of $\text {AUC}_{t}^{(j)}$. Furthermore, it follows that $\sqrt {n}(\text {AUC}_{t}^{(j)}(\hat {\beta }_{j})-\text {AUC}_{t}^{(j)}({\beta }_{0j}))$ converges to a Normal distribution with mean 0 and variance $[\frac {d}{d\beta _{j}}\eta ^{-1}(\textbf {A}\,{\beta _{j}}^{T})]_{\beta _{j}=\hat {\beta }_{j}}^{2}\,\,\textbf {A}\hat {\Sigma }_{j1}^{-1}\hat {\Sigma }_{j2}\hat {\Sigma }_{j1}^{-1}\textbf {A}^{T}$, where $\textbf {A}=(1,t^{p_{1}},t^{p_{2}},\dots,t^{p_{7}}).$

## Results

### Simulation study

Extensive simulation studies are conducted in order to compare the performance of the cWMR, cFPL and semi-parametric [13] estimators for estimating $\text {AUC}^{(j)}_{t}$. We assume two causes of failure (i.e. *j*=1,2) and a baseline marker *M* that is correlated with event time of cause 1, *T*^(1)^ but not with event time of cause 2, *T*^(2)^. We consider several parametric combinations under two major scenarios. For each setting, we generate 500 dataset with a sample size of *n*=500 and for each simulated dataset 200 bootstrap simulations are performed. For each simulation, we estimate $\text {AUC}^{(j)}_{t}$ at predicted time $\text {log}(t)=-1.5,-1.2,\dots,0.6$. We report the average of bootstrap mean estimate of $\text {AUC}^{(j)}_{t}$, absolute relative bias (ARB) of the estimate, average of model based standard error estimate (SE), average of the 500 bootstrap standard errors (BSE) and the coverage probability of the 90% confidence intervals (CI) for the estimates.

#### **Scenario 1**

We assume (log(*T*^(1)^),*M*) jointly follows a bivariate Normal distribution (BVN) with correlation *ρ* i.e. (log(*T*^(1)^),*M*)∼N_2_(*μ*_1_,*μ*_2_,*σ*_1_,*σ*_2_,*ρ*), where *μ*_1_ and *σ*_1_ are mean and standard deviation (SD) of log(*T*^(1)^) and *μ*_2_ and *σ*_2_ are mean and SD of *M*. We show the results for *μ*_1_=0,*μ*_2_=0,*σ*_1_=1,*σ*_2_=1,and *ρ*=−0.7. We consider a negative correlation between the marker and the event time which implies that higher marker value is more indicative of poor survival outcome and hence it is indicative of shorter event time. We further assume log(*T*^(2)^)∼N(0,1) and log(*C*)∼N(0,1), such that approximately 20% subjects are censored. Since *T*^(2)^ and *M* are independent, the I/D ROC curve for the competing cause of failure (i.e. cause 2) lies diagonally on the null ROC curve.

#### **Scenario 2**

We focus on a heterogeneous population where the marginal relationship between *M* and *T*^(1)^ is non-monotone, while *M* and *T*^(2)^ are independent. The heterogeneous population comprised two distinct subgroups (G=0 or 1) and for G=1, *M* is also independent of log(*T*^(1)^). The distribution of (log(*T*^(1)^),*M*) follows a mixture of two BVNs. We show the results for two different parameter combinations:

$$ (a)\,\,\,\, (\text{log}(T^{(1)}), M) \sim \left\{ \begin{array}{cc} \mathrm{N}_{2}(-1.5,-1.5,1,1,0),&\ \mathrm{G}= 1,\\\mathrm{N}_{2}(0,2,1,1,-0.8),&\ \mathrm{G}=0. \end{array}\right. $$

$$(b)\,\,\,\,(\text{log}(T^{(1)}), M) \thicksim \left\{ \begin{array}{cc} \mathrm{N}_{2}(-1.5,2,1,1,0), &\ \mathrm{G}= 1\\ \mathrm{N}_{2}(0,0,1,1,-0.8), &\ \mathrm{G}=0. \end{array}\right. $$

We assume $\mathrm {G}\thicksim $ Bernoulli(0.2). Note that the semi-parametric approach is biased because of violation of monotonicity in this scenario, and therefore not estimated. The resulting $\text {AUC}^{(j)}_{t}$ curves mimic the relationship in the LT data as will be demonstrated later.

In Tables 1, 2, and 3, we summarize the simulation results for scenarios 1–2 respectively. Table 1 demonstrates that the estimates of $\text {AUC}^{(j)}_{t}$ is less biased when derived using cWMR and cFPL compared with the semi-parametric method. For instance, for cause 1, the ARB in the estimate of $\text {AUC}^{(j)}_{t}$for the cWMR is 0.36% corresponding to predicted time log(*t*) = -1.5. However, for the semi-parametric approach this is 2.16%. For large predicted time (i.e. log(*t*) = 0.9) both the cFPL and the semi-parametric estimates show large ARB. The bootstarp standard errors for both cWMR and cFPL methods are close to their corresponding model-based standard error estimates. For cWMR, the coverage probabilities of estimated $\text {AUC}^{(j)}_{t}$ based on 90% estimated confidence intervals are very close to the nominal value of 0.9 across all predicted times. When we have sufficient data around the given predicted times, say -1.5 ≤log(*t*)≤ 0.6, the coverage probabilities of estimated $\text {AUC}^{(j)}_{t}$ using cFPL are very close to the nominal value. However, the coverage probability is much lower than the nominal level when riskset size is 4 at given time log(*t*)=0.9. This is perhaps the issue of oversmoothing. It could be avoided by choosing the large predicted times as the 90th percentile of the observed survival time points [10]. In Tables 2 and 3, we compare the cWMR and cFPL estimates as the semi-parametric estimator is known to be highly biased under scenario 2. Note that, in Table 2, the AUC value for cause 1 increases steadily between −1.5≤ log(*t*) ≤ 0 and then start decreasing. This kind of non-monotone pattern may be due to violation of monotone relationship between marker and event time. The estimates of $\text {AUC}^{(j)}_{t}$ for both cWMR and cFPL are close to their corresponding true values when the predicted time is small. In these comparisons cFPL performs slightly better than cWMR. The cFPL method yields substantially greater variances for large values of log(*t*) compared to the cWMR. For both methods, the estimated coverage probabilities are very close to the nominal coverage probability of 90% except for edges.

**Table 1:** Simulation results for estimation of incident/dynamic cause specific AUC of a single marker in Scenario 1, comparing the methods cWMR, Semi-parametric and cFPL

				cWMR				Semi-parametric					cFPL				
Cause	Log(t)	$\sum _{i=1}^{i=n}R_{i}(t)$	AUC	Mean	ARB	BSE	SE	CP	Mean	ARB	BSE	CP	Mean	ARB	BSE	SE	CP
1	-1.5	407	0.833	0.830	0.36	0.031	0.030	88.0	0.815	2.16	0.046	90.0	0.833	0	0.026	0.028	93.1
	-1.2	347	0.802	0.800	0.25	0.030	0.029	89.0	0.783	2.37	0.050	89.6	0.802	0	0.025	0.027	93.5
	-0.9	272	0.771	0.771	0	0.030	0.029	84.1	0.753	2.33	0.053	89.6	0.771	0	0.026	0.028	92.1
	-0.6	192	0.743	0.743	0	0.032	0.031	87.3	0.727	2.15	0.055	93.4	0.742	0.1	0.030	0.031	91.3
	-0.3	119	0.716	0.719	0.41	0.038	0.036	88.6	0.707	1.25	0.057	90.0	0.716	0	0.036	0.038	93.1
	0.0	63	0.693	0.696	0.43	0.048	0.046	86.9	0.684	1.23	0.058	90.2	0.694	0.14	0.053	0.050	92.8
	0.3	28	0.672	0.676	0.6	0.071	0.064	89.7	0.670	0.29	0.056	89.0	0.678	0.89	0.094	0.084	93.1
	0.6	10	0.654	0.659	0.76	0.126	0.104	86.0	0.653	0.15	0.060	92.6	0.653	0.15	0.211	0.186	87.8
	0.9	4	0.639	0.657	2.91	0.189	0.169	89.0	0.578	9.54	0.119	82.1	0.599	6.10	0.353	0.586	75.6
2	-1.5	407	0.5	0.501	0.2	0.051	0.050	87.3	0.504	0.8	0.082	90.2	0.5	0	0.045	0.049	92.1
	-1.2	347	0.5	0.501	0.2	0.045	0.044	88.5	0.503	0.6	0.078	91.2	0.5	0	0.040	0.042	91.9
	-0.9	272	0.5	0.501	0.2	0.041	0.041	88.8	0.499	0.4	0.075	90.0	0.499	0.2	0.037	0.04	92.1
	-0.6	192	0.5	0.500	0.0	0.042	0.041	89.1	0.501	0.4	0.071	86.8	0.499	0.2	0.041	0.041	90.9
	-0.3	119	0.5	0.499	0.2	0.047	0.045	90.5	0.494	1.2	0.069	90.2	0.498	0.4	0.047	0.049	93.2
	0.0	63	0.5	0.499	0.2	0.057	0.054	86.3	0.496	0.8	0.065	91.4	0.498	0.4	0.068	0.061	91.3
	0.3	28	0.5	0.499	0.2	0.080	0.073	87.3	0.505	1.0	0.063	90.4	0.496	0.8	0.120	0.107	92.5
	0.6	10	0.5	0.500	0.0	0.136	0.111	83.3	0.496	1.6	0.062	89.2	0.498	0.4	0.247	0.223	87.7
	0.9	4	0.5	0.499	0.2	0.209	0.191	87.1	0.471	5.8	0.103	92.0	0.495	1.0	0.376	0.497	62.8

Average of bootstrap mean (Mean), average of bootstrap standard errors (BSE), absolute relative bias (ARB), model-based standard error (SE), coverage probability (CP)(nominal level is 90 percentage) of AUC

**Table 2:** Simulation results for estimation of incident/dynamic cause-specific AUC of a single marker in Scenario 2(*a*), comparing the methods- cWMR & cFPL

			cWMR				cFPL					
Cause	Log(t)	AUC	Mean	ARB	BSE	SE	CP(%)	Mean	ARB	BSE	SE	CP(%)
1	-1.5	0.535	0.538	0.561	0.061	0.052	83.6	0.534	0.187	0.047	0.051	92.2
	-1.2	0.600	0.598	0.333	0.054	0.049	86.0	0.597	0.500	0.043	0.048	93.5
	-0.9	0.649	0.643	0.924	0.049	0.047	90.3	0.645	0.616	0.042	0.043	93.5
	-0.6	0.680	0.674	0.882	0.047	0.047	90.5	0.678	0.294	0.043	0.046	93.0
	-0.3	0.692	0.690	0.289	0.049	0.050	92.7	0.693	0.144	0.052	0.05	89.4
	0.0	0.697	0.698	0.143	0.057	0.057	89.2	0.697	0.00	0.080	0.069	92.6
	0.3	0.688	0.697	1.308	0.080	0.073	87.0	0.702	2.034	0.139	0.128	92.6
	0.6	0.681	0.695	2.056	0.135	0.113	83.0	0.655	3.818	0.284	0.294	83.2
2	-1.5	0.5	0.5000	0.0	0.049	0.052	88.5	0.5005	0.1	0.048	0.052	90.7
	-1.2	0.5	0.4999	0.02	0.043	0.046	87.5	0.4988	0.24	0.042	0.044	92.7
	-0.9	0.5	0.4974	0.52	0.040	0.043	88.0	0.4979	0.42	0.040	0.044	92.3
	-0.6	0.5	0.4976	0.48	0.041	0.044	90.0	0.4975	0.50	0.044	0.044	91.8
	-0.3	0.5	0.4985	0.30	0.046	0.049	89.7	0.4973	0.54	0.052	0.054	94.0
	0.0	0.5	0.4968	0.64	0.057	0.049	89.9	0.4967	0.66	0.079	0.068	92.1
	0.3	0.5	0.4952	0.96	0.080	0.080	87.2	0.4953	0.94	0.143	0.128	94.2
	0.6	0.5	0.5011	0.22	0.137	0.124	83.4	0.4969	0.62	0.269	0.289	87.8

Average of bootstrap mean (Mean), average of bootstrap standard errors e relative bias (ARB(%)), model-based standard error (SE), and coverage probability (CP(%))-(nominal level —90%) of AUC

**Table 3:** Simulation results for estimation of incident/dynamic cause-specific AUC of a single marker in Scenario 2(*b*), comparing the methods- cWMR & cFPL

			cWMR				cFPL					
Cause	Log(t)	AUC	Mean	ARB	BSE	SE	CP(%)	Mean	ARB	BSE	SE	CP(%)
1	-1.5	0.843	0.843	0.0	0.022	0.025	92.0	0.843	0.0	0.017	0.018	93.3
	-1.2	0.827	0.830	0.31	0.023	0.026	94.0	0.829	0.245	0.018	0.02	92.3
	-0.9	0.807	0.809	0.26	0.025	0.028	93.8	0.810	0.042	0.020	0.021	89.9
	-0.6	0.784	0.786	0.25	0.029	0.031	94.8	0.788	0.451	0.025	0.026	93.6
	-0.3	0.760	0.765	0.62	0.036	0.038	94.0	0.761	0.162	0.033	0.033	92.9
	0.0	0.737	0.747	1.41	0.048	0.049	90.8	0.737	0.016	0.054	0.046	92.7
	0.3	0.716	0.727	1.60	0.073	0.70	88.0	0.728	1.66	0.096	0.085	89.7
	0.6	0.696	0.710	2.07	0.131	0.110	83.9	0.692	0.46	0.218	0.179	84.6
2	-1.5	0.5	0.5029	0.58	0.049	0.052	88.9	0.5028	0.56	0.048	0.0517	92.3
	-1.2	0.5	0.5017	0.34	0.043	0.046	90.0	0.5025	0.50	0.042	0.45	94.3
	-0.9	0.5	0.5004	0.08	0.040	0.043	89.9	0.5003	0.06	0.040	0.044	93.7
	-0.6	0.5	0.4987	0.26	0.042	0.044	88.7	0.4983	0.34	0.045	0.044	92.1
	-0.3	0.5	0.5005	0.10	0.046	0.049	93.1	0.4991	0.178	0.052	0.054	93.5
	0.0	0.5	0.5006	0.12	0.057	0.06	90.5	0.5030	0.60	0.080	0.07	92.5
	0.3	0.5	0.5043	0.86	0.082	0.081	89.3	0.5039	0.78	0.146	0.126	95.1
	0.6	0.5	0.5042	0.84	0.142	0.124	87.5	0.4938	0.62	0.276	0.259	85.0

Average of bootstrap mean (Mean), average of bootstrap standard errors (BSE), absolute relative bias (ARB(%)), model-based standard error (SE) and coverage probability (CP(%))-(nominal level —90%) of AUC

Overall, our results demonstrate that both cWMR and cFPL appear to perform well compared to semi-parametric approach in terms of bias and standard errors. In addition, in scenario 2 where the monotonicity between marker and event time is violated, the semi-parametric approach is known to be biased. However, both our proposed methods perform adequately well.

### Application

**Fig. 1:**
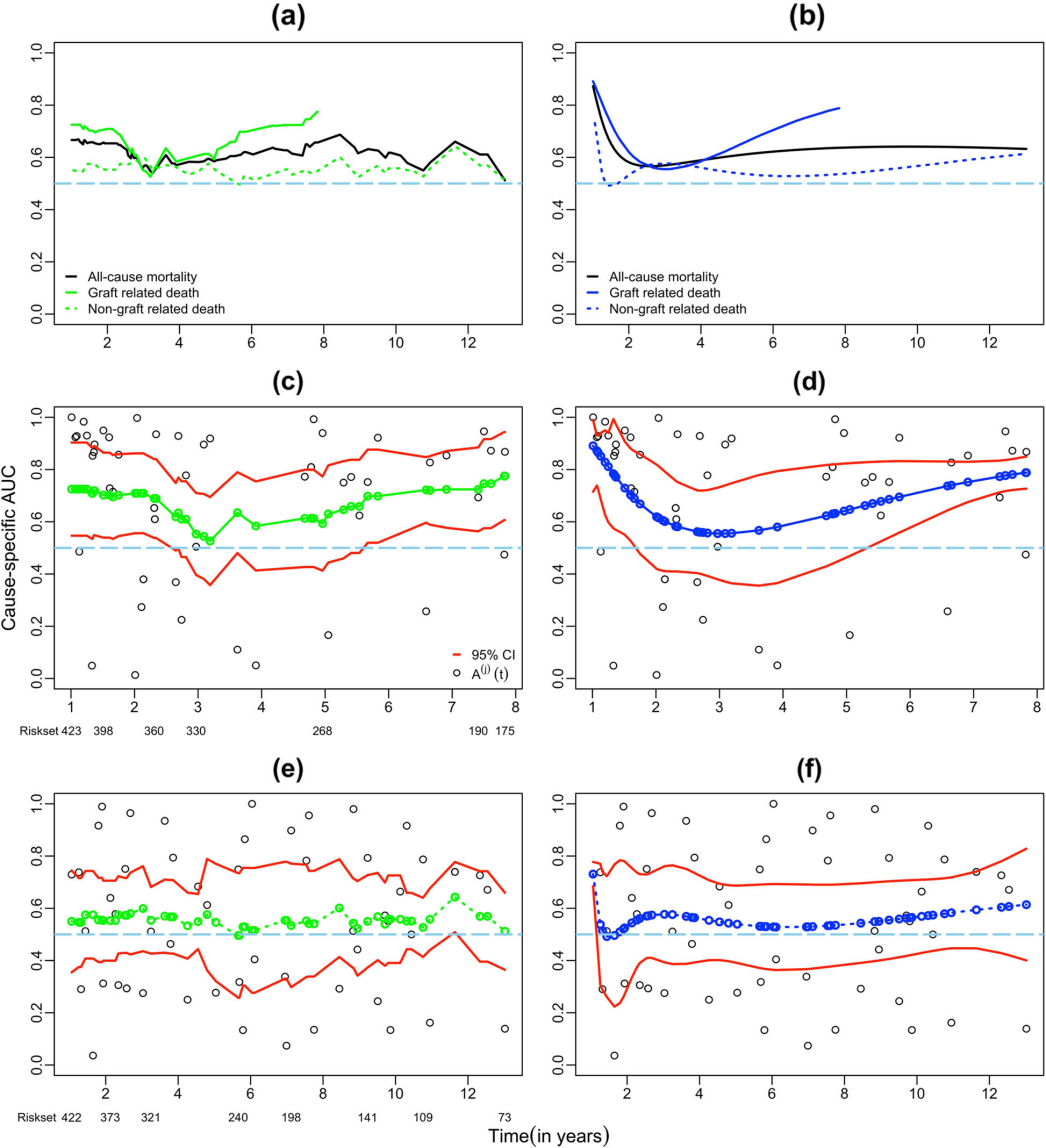
Estimates of incident/dynamic cause-specific AUC(*t*) curves using weighted mean rank (cWMR) and fractional polynomial (cFPL) for Liver Transplantation data. Plots (*a*) and (*b*) illustrate the incident/dynamic AUC(*t*) curve for all-cause mortality, incident/dynamic cause-specific AUC(*t*) curves for graft-related death and non-graft-related death. Plots (*a*) and (*b*) are estimated using cWMR and cFPL methods, respectively. Plots (*c*) and (*d*) illustrate the incident/dynamic cause-specific AUC(*t*) curves for graft-related death with pointwise 95% confidence intervals (CI) using cWMR and cFPL methods, respectively. Plots (*e*) and (*f*) illustrate the incident/dynamic cause-specific AUC(*t*) curves for non-graft-related death with pointwise 95% CIs using cWMR and cFPL methods, respectively

We demonstrate the proposed methods for estimating $\text {AUC}^{(j)}_{t}$ using LT data from a retrospective study conducted at the McGill University Health Center [3]. The LT study included 547 patients who underwent LT between 1991 and 2012 and who met the criteria: patient with graft survival >12 months; serum fibrosis biomarkers including FIB-4 and NAFLD score available at 1 year after LT; and a minimum follow-up of 1 year. The study found that serum fibrosis markers performed well in predicting death and graft loss in LT recipients. According to the authors, this is the first study to establish the prognostic value of the fibrosis markers in a large cohort of LT recipients over a long-term follow-up period. We further analyzed a subset of the subjects (*n*=423) after excluding subjects with missing outcome and/or marker values. During the study period, 64 patients who underwent LT died due to graft-related causes (e.g. graft failure). However, 62 patients died of causes that are unrelated to their transplantation (e.g. sepsis, cardiovascular disease, renal, respiratory failure etc.). Different causes of death led to a competing risks situation. The research objective is to evaluate the performance of FIB-4 as a marker to discriminate between subjects who died due to graft related causes and those who died of non-graft related causes after the LT. The top two subplots in Figure 1 show the estimated AUC obtained using cWMR and cFPL methods for graft-related, non-graft related and all-cause death (considering both graft and non-graft related death as events of interest). Irrespective of the methods, the AUC curve for all-cause mortality is biased compared to the curves for graft and no-graft related deaths. The magnitude of bias is downward compared to graft related deaths and the bias is upward compared to non-graft related deaths. It indicates that consideration of all-cause mortality as an event of interest instead of competing risks will result in biased accuracy estimates. Therefore, it leads us to analyse the LT data using the methodology proposed here in the presence of competing risks. Figure 1 also illustrates estimated $\text {AUC}^{(j)}_{t}$ curves with 95% CI for graft and non-graft related causes using cWMR and cFPL methods. In order to estimate $\text {AUC}^{(j)}_{t}$ using cWMR, the choice of neighborhood was 1.35 years back and forward. This bandwidth of 1.35 years is obtained after minimizing the IMSE. The estimated $\text {AUC}^{(j)}_{t}$ curves of the FIB-4 for the graft related death sustains a high predictive value (above 0.65 for most of the study duration) irrespective of the estimation procedures. For example, over the first 3 years of follow-up the estimated $\text {AUC}^{(j)}_{t}$ under cFPL approach ranges between 0.89 to 0.56. This implies that on any day, *t*, during the first 3 years of follow-up, the probability that a subject after LT who dies due to graft related causes on day *t* having a FIB-4 value greater than a subject who survives beyond day *t* is at least 0.56. Overall, $\text {AUC}^{(j)}_{t}$ curve estimated using cFPL is a smooth function over predicted time while estimated $\text {AUC}^{(j)}_{t}$ curve of cWMR is less smooth. We could not find any definitive reasons for the cause-specific AUC(*t*) for graft related cause increases between years 3.3 and 8. However, in reference to results from simulation scenario 2, we observed that if there is any latent (or unobserved) heterogeneity in the data, the AUC curve shows non-monotone trend over time. On the other hand, these two curves for non-graft related death are almost flat around the horizontal line at AUC(*t*) = 0.5. Furthermore, 95% CI’s of the estimates of AUC contain the null value of 0.5 which implies that FIB-4 is non-informative as a prognostic marker for non-graft related events. Therefore, FIB-4 as a baseline marker does not discriminate patients with non-graft related deaths after LT, which is expected. In addition, under cFPL method the CIs of the estimates of $\text {AUC}^{(j)}_{t}$ curves at the tails of the study period are relatively wider/narrower than that of under cWMR method. The CIs of the estimates using cFPL are wider in both small and large predicted times because cFPL may have oversmoothing issue especially towards the start and end of the study. Finally, our analysis indicates a better discrimination by FIB-4 for graft related deaths than non-graft related deaths after LT for most of the study duration.

## Discussion

Measures of calibration and discrimination are integral parts to evaluate prognostic accuracy of a marker. Calibration indices provide information on how close the predicted risks are to the observed risks while discrimination indices measure whether markers correctly discriminate subjects who will subsequently experience the event of interest at or by time *t* from those who will not experience any event by *t*. Calibration measures e.g. an expected Brier score for competing risks and corresponding estimator were provided in [17]. It measures the closeness of the observed event status and the model predicted event probabilities in the presence of competing risks. Here, we primarily focus on the discrimination accuracy of marker. The main goal of this manuscript is to estimate cause-specific AUC(*t*) of a baseline marker in the presence of competing risks. During such estimation, analysts often censor subjects when a competing event occurs. For instance, the outcome in LT study is time-to-death attributable to LT-related causes, an analyst may consider a subject as censored once that subject dies of causes unrelated to LT. Because subjects who died of non LT-related causes are not at risk of dying due to LT, censoring these competing risks events (informative censoring) may lead to distorted risk estimates [5] and subsequently biased accuracy estimators. Alternatively, some may consider a composite event where deaths attributable to LT and non-LT deaths are merged together as any adverse events. In the “Application” section, we mimic this to our LT data to show the drawback of using a composite endpoint to demonstrate the importance of considering competing risks in prognostic accuracy estimation. It is evident from our results that simply considering a composite event instead of competing events introduces bias in accuracy estimation. For competing risks analysis, the influence of covariate can be evaluated in relation to cause-specific hazard or the sub-distribution hazard of different causes of failure. For estimating cause-specific hazard, when a subject experience any event, they are removed from the subsequent risksets. In contrast, for estimating sub-distribution hazard [18], a subject who experiences a competing event is not removed from the riskset at that time, but rather is censored at the end of the follow-up.

We propose a local and a global estimators of cause-specific AUC(*t*) for right-censored survival time outcome in the presence of competing risks. In [13], a semi-parametric approach based on Cox model was suggested for estimating cause-specific AUC(*t*). This approach provides bias estimate when the association between event time and marker is non-monotone. Also, this is not a one-step approach for estimating AUC trajectory over time and it requires longer computation time. In addition, their method lacks analytical development of the large sample properties for statistical inference. These motivate us to propose new estimators for estimating cause-specific AUC(*t*). As pointed out earlier, the observed proportion of controls ranked lower than the (cause-specific) case, generally leads to unstable estimates because it is based on a single ’case’ subject who had an event of interest at the specific time. Hence, the estimation of cause-specific AUC that is based on observed proportions requires some degree of smoothing. Our proposed estimators implement the degree of smoothing in different ways. The local estimator - cause-specific weighted mean rank (cWMR) - is a local average of unsmoothed time specific observed cause-specific AUCs within a neighborhood of a given time *t*. cWMR is sensitive to neighborhood span. The width of the neighbourhood directly influenced the smoothness of the curves especially towards the end of the study when size of the riskset gets very small. We have considered cross-validation approach to choose optimal neighborhood span. Use of adaptive smoothing techniques, for example, fixing the number of neighbors instead of a fixed bandwidth may be useful. Instead of using local average of concordance within a neighborhood of time, we propose an alternative method based on global curve fitting approach, cFPL, which estimates the cause-specific AUC as a function of time through a flexible fractional polynomial function. It expresses the unsmoothed intermediate AUCs as a function of fractional polynomials of time and then estimates the coefficients of polynomials through a partial likelihood optimization. cFPL overcomes the issue of sensitivity to neighborhood span of cWMR. However, oversmoothing is an issue with cFPL in both small and large time points. In terms of computation time, cWMR is computationally efficient compared to cFPL. We also develop the large sample properties of both estimators as well as their corresponding analytical variances. Our simulation study suggests that these two estimators perform very well compared to the existing semi-parametric method for measuring cause-specific AUC. The performance has been evaluated in terms of absolute relative bias and coverage probability. Between our two proposed approaches, both estimators show similar level of relative bias particularly for small predicted time in simulation studies. However, for large times the cFPL estimator shows large bias compared to the cWMR estimator. In addition, the coverage probability of cFPL estimator is very different from the nominal coverage probability particularly at the edges. In LT study, our goal is to evaluate the performance of FIB-4 as a baseline biomarker to discriminate between subjects who died due to graft related causes against those who died of non-graft related causes after the liver transplantation. Our analysis indicates a better discrimination of graft related deaths than non-graft related deaths after LT for most of the study duration. The estimation methods assume that the censoring time is independent of the survival time. It warrants additional research to allow covariate dependent censoring. Furthermore, in our settings, we do not consider time-varying marker. However, the proposed methodology is equally applicable to settings where time-varying marker exist.

Finally, between two estimators, which one is better: cWMR and cFPL? There is no general answer. The former is more adaptive to the local changes while the latter is good for an overall description. Our advice is to perform sensitivity analysis in which the choice of estimation methods vary. Also, for all methods the estimates at the higher time range are unstable, emphasizing the fact that one should have a sufficient number of events for estimation of the cause-specific AUC. This problem may be avoided by choosing a sufficiently wide neighborhood (e.g. fixing the number of neighbors) when using the cWMR. For cFPL, we can choose relevant short future time horizon over which we have sufficient cause-specific cases for numerically stable results.

Our study has some limitations. The first limitation is related to the bandwidth selection in cWMR approach. To implement the proposed cWMR estimator in practice, the appropriate bandwidths must be chosen. In this manuscript, we have used leave-one-out cross validation approach [9]. It would be interesting to perform sensitivity analysis by varying different bandwidth selectors. Next, our proposed approaches are not applicable when the data have missing information. However, following a typical imputation method, our approach could be applied to the imputed data. Details of the inference and sensitivity to the imputation methods is yet to be explored. Furthermore, the variance calculation of cFPL estimator is computationally intensive. Future research to explore alternate methods for efficient computation may be worthwhile. Moreover, the estimation methods assume that the censoring time is independent of the survival time. Additional research to allow covariate dependent censoring is warranted. In addition, in our settings, we do not consider time-varying marker. However, the proposed methodology is equally applicable to settings where time-varying marker exist. This is to be explored in the future.

## Conclusions

We developed estimation procedures of estimating time-dependent prognostic accuracy measures for a right-censored time-to-event outcome in the presence of competing risks. The proposed methods are non-parametric, direct and computationally simple that will overcome the shortcomings of the existing approach. The methods will provide computationally efficient options for assessing the prognostic accuracy of markers for time-to-event outcome.

## Additional material

Additional file 1 This file includes the proof of asymptotic properties of cWMR and cFPL estimators and their corresponding variance estimation techniques.

Additional file 2 This file includes the computer code (written in R programming language) for estimating cause-specific AUC (*t*) using cWMR and cFPL estimators.

Additional file 3 this file includes description files for using r code provided in Additional file 2 for estimating cause-specific aUC(*t*) using cWMR and cFPL estimators.

## Data Availability

The liver transplant dataset is not publicly available due to patient privacy restrictions, but could be made available from the corresponding author pending appropriate approval. The R codes for simulations and data analysis are provided in additional files.

## Abbreviations

KDRI: Kidney donor risk index
LT: liver-transplantation
FIB-4: fibrosis score 4
NAFLD: nonalcoholic fatty liver disease fibrosis score
ROC: Receiver operating characteristic curve
AUC: Area under the ROC curve
I: Incident cases
D: Dynamic Controls
C: Cumulative cases
WMR: Weighted Mean Rank
FPL: Fractional Polynomial
cWMR: Weighted Mean Rank Estimator of I/D Cause-Specific AUC(t)
cFPL: Fractional Polynomial Estimator of I/D Cause-Specific AUC(t)
BVN: bivariate Normal distribution
ARB: absolute relative bias
BSE: bootstrap standard errors
CI: Confidence interval
SD: standard deviation
