# Supplementary material for: Inference about time-dependent prognostic accuracy measures in the presence of competing risks
Source: BMC Med Res Methodol. 2020 Aug 28;20:219. doi: 10.1186/s12874-020-01100-0 (PMC7456384; doi:10.1186/s12874-020-01100-0)
Supplement: Supplementary file 1 — This file includes the proof of asymptotic properties of cWMR and cFPL estimators and their corresponding variance estimation techniques. [file 12874_2020_1100_MOESM1_ESM.pdf]

# Additional File 1: Inference about Time-dependent Prognostic Accuracy Measures in the Presence of Competing Risks

## Appendix

### Proof of Theorem 1

We restrict our attention to a neighborhood around $t$ and cause $j$ i.e. $N_t^{(j)}(h_n) = \{t_k : |t - t_k| < h_n, \delta_k = j\}$ with size $|N_t^{(j)}(h_n)| = m_t^{(j)}$. Other causes can be treated in a similar way. For simplicity, we assume that no subjects are censored. Let $B_t$ denote the number of subjects at the start of the neighborhood i.e. at time $t-h_n$. We assume that no failures other than cause $j$ within neighborhood. We also assume no two subjects fail at the same time. The ordered unique event time for $i$-th subject is $T_i = t_{(i)}$. The indices of the observed event times due to cause $j$ within the neighborhood $N_t^{(j)}(h_n)$ are then $B_t{+}1, B_t{+}2, ..., B_t{+}m_t^{(j)}$ and that $t_{(B_t+1)}, t_{(B_t+2)}, ..., t_{(B_t+m_t^{(j)})}$ are the corresponding ordered unique event times within $N_t^{(j)}(h_n)$. The $j$-th cause-specific weighted mean rank estimator can be written as

$$\begin{aligned}
\mathrm{cWMR}_t^{(j)} &= \frac{1}{|N_t^{(j)}(h_n)|} \sum_{t_{(i)} \in N_t^{(j)}(h_n)} \mathrm{A}^{(j)}(t_{(i)}), \\
&= \frac{1}{m_t^{(j)}} \sum_{i=B_t+1}^{B_t+m_t^{(j)}} \mathrm{A}^{(j)}(t_{(i)}), \\
&= \frac{1}{m_t^{(j)}} \sum_{i=B_t+1}^{B_t+m_t^{(j)}} \frac{1}{n_{t_{(i)}}} \sum_{k=i+1}^{n} \mathbf{1}\{M_i > M_k\}\ \mathbf{1}\{T_i < T_k\}\ \mathbf{1}\{\delta_i = j\}, \\
&= \sum_{i \neq k} \frac{1}{2 \times m_t^{(j)} \times n_{t_{(i)}}} \mathbf{1}\{M_i > M_k\}\ \mathbf{1}\{T_i < T_k\}\ \mathbf{1}\{\delta_i = j\}
\end{aligned}$$

From the above representation of $\mathrm{cWMR}_t^{(j)}$, it can be seen that $\mathrm{cWMR}_t^{(j)}$ is a linear transformation of weighted U-statistic i.e. $\mathrm{cWMR}_t^{(j)} = 2\ (U^{(j)} + 1)$ where

$$U^{(j)} = \sum_{i \neq k} w_{ik}^{(j)}\ \mathbf{1}\{k > i\}\ \mathbf{1}\{R_i > R_k\}\ \mathbf{1}\{\delta_i = j\},$$

where $R_i$ is the rank of the marker $M$ corresponding to the $i$-th ordered observed failure time and $w_{ik}^{(j)} = \frac{1}{2\times m_t^{(j)} \times n_{t_{(i)}}}$. According to [1], $W_n = \sum_{n,k} W(S)\phi(S)$ be a weighted U-statistic of order 2 with $w_{i,n} = \sum_{i \epsilon S} w(S)$ and $W_n^* = \sum_{i=1}^n w_{i,n}\psi_i(X_i)$. Suppose that, as $n \to \infty$, the following conditions hold

1. $\dfrac{\max\limits_{1\le i\le n} |w_{i,n}|^2}{\sum_i w_{i,n}^2} \to 0,$
2. $\dfrac{\sum_{(n,2)} w^2(S)}{\sum_i w_{i,n}^2} \to 0$ and
3. $E|\psi_i(X_i)|^{(2+\delta)} \le \infty$ for some $\delta > 0$.

Then

$$\frac{(W_n - \theta)}{\sqrt{Var(W_n)}} \xrightarrow{D} N(0,1).$$

To prove asymptotic Normality of cWMR$_t^{(j)}$ we need to verify the conditions (1)-(3). Similar to the arguments and derivation in [2], we will verify them. To simplify notations we will drop the subscript $n$ from $w_{i,n}^{(j)}$. Let $w_i^{(j)}$ denote as

$$\begin{aligned} w_i^{(j)} &= \sum_k w_{ik}^{(j)}, \\ &= \begin{cases} \dfrac{1}{2\times m_t^{(j)}}(1 + \sum\limits_{k<i} \dfrac{1}{|R_{0t_{(k)}}|}) & i = B_t + 1, \ldots, B_t + m_t^{(j)}, \\ \dfrac{1}{2\times m_t^{(j)}} \sum\limits_k \dfrac{1}{|R_{0t_{(k)}}|} & i > B_t + m_t^{(j)}. \end{cases} \end{aligned}$$

To prove condition (1), let's begin with

$$\begin{aligned} \max_{B_t+1 \le i \le n} |w_i^{(j)}|^2 = [w_{B_t+m_t^{(j)}}^{(j)}]^2 &= \sum_{k=B_t}^{n} [w_{B_t+m_t^{(j)},k}^{(j)}]^2 \\ &= \frac{1}{4\times [m_t^{(j)}]^2}(1 + \frac{1}{n_{B_t+1}} + \frac{1}{n_{B_t+2}} + \cdots + \frac{1}{n_{B_t+m_t^{(j)}-1}})^2 \\ &\le \frac{1}{4\times [m_t^{(j)}]^2}(1 + \frac{m_t^{(j)}}{n_{B_t+m_t^{(j)}}})^2 \end{aligned}$$

Again,

$$
\begin{aligned}
4 \times [m_t^{(j)}]^2 \times \sum_i [w_i^{(j)}]^2 &= 1 + (1 + \frac{1}{n_{B_t+1}})^2 + (1 + \frac{1}{n_{B_t+1}} + \frac{1}{n_{B_t+2}})^2 + \dots \\
&+ (1 + \frac{1}{n_{B_t+1}} + \frac{1}{n_{B_t+2}} + \dots + \frac{1}{n_{B_t+m_t^{(j)}-1}})^2 \\
&+ (n - B_t + m_t^{(j)}) \times (\frac{1}{n_{B_t+1}} + \frac{1}{n_{B_t+2}} + \dots + \frac{1}{n_{B_t+m_t^{(j)}-1}} + \frac{1}{n_{B_t+m_t^{(j)}}})^2 \\
&\geq (n - B_t + m_t^{(j)})(\frac{m_t^{(j)}}{n_{B_t+1}})^2
\end{aligned}
$$

If $n \to \infty$ then $(n - B_t + m_t^{(j)}) \to \infty$, therefore

$$
\frac{\max_{B_t+1 \leq i \leq n} |w_i^{(j)}|^2}{\sum_i [w_i^{(j)}]^2} \leq \frac{(1 + \frac{m_t^{(j)}}{n_{B_t+m_t^{(j)}}})^2}{(n - B_t + m_t^{(j)})[\frac{d_t^{(j)}}{n_{B_t+1}}]^2} \to 0,
$$

condition (1) is satisfied. Again, for condition (2) we are following the algebraic arguments provided on page 16 in [2], and therefore, we can write

$$
4 \times [m_t^{(j)}]^2 \sum_{k \neq i} [w_{ik}^{(j)}]^2 \leq \frac{2 \times m_t^{(j)}}{n_{B_t+m_t^{(j)}}}.
$$

Also,

$$
\frac{\sum_{k \neq i} [w_{ik}^{(j)}]^2}{\sum_i [w_i^{(j)}]^2} \leq \frac{\frac{2 \times m_t^{(j)}}{n_{B_t+m_t^{(j)}}}}{(n - B_t + m_t^{(j)})[\frac{m_t^{(j)}}{n_{B_t+1}}]^2} \to 0 \text{ as } n \to \infty
$$

and hence condition (2) is satisfied. Again, for condition (3), let $\psi(X_i, X_j) = \mathbf{1}\{k > i\}\ \mathbf{1}\{R_i > R_k\}\mathbf{1}\{\delta_i = j\}$. The expectation of $\psi(X_i, X_j)$, $E|\psi(X_i, X_j)| \leq \infty$ since $|\psi(X_i, X_j)| \leq 1$. So, $E|\psi(X_i, X_j)|^{(2+\delta)} \leq \infty$ for some $\delta > 0$.

## Estimation of variance of cWMR$_t^{(j)}$

The variance of cWMR$_t^{(j)}$,V$_n^{(j)}$, is written as

$$
\mathrm{V}_n^{(j)} = \frac{1}{[m_t^{(j)}]^2}[\sum_{t_{(i)} \in N_t^{(j)}(h_n)} \mathrm{var}[\mathrm{A}^{(j)}(t_{(i)})] + \sum_{t_{(i)} \neq t_{(k)}} \mathrm{cov}[\mathrm{A}^{(j)}(t_{(i)}), \mathrm{A}^{(j)}(t_{(k)})]],
$$

In order to compute $\mathrm{var}[\mathrm{A}^{(j)}(t_{(i)})]$ and $\mathrm{cov}[\mathrm{A}^{(j)}(t_{(i)}), \mathrm{A}^{(j)}(t_{(k)})]$ we propose the following estimators in the spirit of variance calculation of AUC proposed in [3]

and [2]

$$\text{var}[\mathrm{A}^{(j)}(t)] = (\frac{n_t - 1}{n_t})\{P_1^{(j)}(t) - [P_0^{(j)}(t)]^2\} + \frac{1}{n_t}\{P_0^{(j)}(t)(1 - P_0^{(j)}(t))\},$$

$$\text{cov}[\mathrm{A}^{(j)}(t), \mathrm{A}^{(j)}(s)] = \frac{1}{n_t}[\{P_2^{(j)}(t,s) - P_{2.0}^{(j)}(t,s)\} + \{P_3^{(j)}(t,s) - P_{3.0}^{(j)}(t,s)\}],$$

where

$$\mathrm{P}_0^{(j)}(t) = \Pr(M_i > M_k \mid T_i = t, \delta_i = j, T_k > t),$$

$$\mathrm{P}_1^{(j)}(t) = \Pr(M_i > M_k,\ M_i > M_{k_1} \mid T_i = t, \delta_i = j, T_k > t, T_{k_1} > t),$$

$$\mathrm{P}_2^{(j)}(t,s) = \Pr(M_i > M_k,\ M_l > M_k \mid T_i = t, \delta_i = j, T_l = s, \delta_l = j, T_k > s),$$

$$\mathrm{P}_{2.0}^{(j)}(t,s) = \Pr(M_i > M_k \mid T_i = t, \delta_i = j, T_k > s) \times \Pr(M_l > M_k \mid T_l = s, \delta_l = j, T_k > s)$$

$$\mathrm{P}_3^{(j)}(t,s) = \Pr(M_i > M_l,\ M_l > M_k \mid T_i = t, \delta_i = j, T_l = s, \delta_l = j, T_k > s),$$

$$\mathrm{P}_{3.0}^{(j)}(t,s) = \Pr(M_i > M_l \mid T_i = t, \delta_i = j, T_l = s, \delta_l = j) \times \Pr(M_l > M_k \mid T_l = s, \delta_l = j, T_k > s),$$

To estimate $P_0^{(j)}(.), P_1^{(j)}(.)$ etc. we use a Normal approximation for the case due to $j$-th cause and control markers after a rank-based Z-score transformation and then empirically estimating the parameters of the approximating Normal distributions. Let $\mu_1^{(j)}(t)$ and $\sigma_1^{(j)}(t)$ can be estimated as the mean and variance of the marker of the $j$-the cause-specific cases at $t$. While $\mu_0(t)$ and $\sigma_0(t)$ can be estimated as the mean and variance of the marker of the controls at $t$.

$$\hat{\mathrm{P}}_0^{(j)}(t) = \phi(\frac{\mu_1^{(j)}(t) - \mu_0(t)}{\sqrt{(\sigma_1^{(j)}(t))^2 + \sigma_0^2(t)}}),$$

$$\hat{\mathrm{P}}_1^{(j)}(t) = \phi_2(\mathbf{0}|\mu_2, \Sigma_2),$$

where

$$\mu_2 = \begin{pmatrix} \mu_0(t) - \mu_1^{(j)}(t) \\ \mu_0(t) - \mu_1^{(j)}(t) \end{pmatrix}, \quad \Sigma_2 = \begin{pmatrix} (\sigma_1^{(j)}(t))^2 + \sigma_0^2(t) & (\sigma_1^{(j)}(t))^2 \\ (\sigma_1^{(j)}(t))^2 & (\sigma_1^{(j)}(t))^2 + \sigma_0^2(t) \end{pmatrix}.$$

$$\hat{\mathrm{P}}_2^{(j)}(t,s) = \phi_2(\mathbf{0}|\mu_3, \Sigma_3),$$

where

$$\mu_3 = \begin{pmatrix} \mu_0(s) - \mu_1^{(j)}(t) \\ \mu_0(s) - \mu_1^{(j)}(s) \end{pmatrix}, \quad \Sigma_3 = \begin{pmatrix} (\sigma_1^{(j)}(t))^2 + \sigma_0^2(s) & \sigma_0^2(s) \\ \sigma_0^2(s) & (\sigma_1^{(j)}(s))^2 + \sigma_0^2(s) \end{pmatrix}.$$

$$\hat{\mathrm{P}}_{2.0}^{(j)}(t,s) = \phi(\frac{\mu_1^{(j)}(t)-\mu_0(s)}{\sqrt{(\sigma_1^{(j)}(t))^2+\sigma_0^2(s)}}) \times \phi(\frac{\mu_1^{(j)}(s)-\mu_0(s)}{\sqrt{(\sigma_1^{(j)}(s))^2+\sigma_0^2(s)}}),$$

$$\hat{\mathrm{P}}_{3}^{(j)}(t,s) = \phi_2(\mathbf{0}|\mu_4, \Sigma_4),$$

where

$$\mu_4 = \begin{pmatrix} \mu_1^{(j)}(s)-\mu_1^{(j)}(t) \\ \mu_0(t)-\mu_1^{(j)}(s) \end{pmatrix}, \quad \Sigma_4 = \begin{pmatrix} (\sigma_1^{(j)}(s))^2+(\sigma_1^{(j)}(t))^2 & -\sigma_0^2(s) \\ -\sigma_0^2(s) & (\sigma_1^{(j)}(s))^2+\sigma_0^2(s) \end{pmatrix}$$

$$\hat{\mathrm{P}}_{3.0}^{(j)}(t,s) = \phi(\frac{\mu_1^{(j)}(t)-\mu_1^{(j)}(s)}{\sqrt{(\sigma_1^{(j)}(t))^2+(\sigma_1^{(j)}(s))^2}}) \times \phi(\frac{\mu_1^{(j)}(s)-\mu_0(s)}{\sqrt{(\sigma_1^{(j)}(s))^2+\sigma_0^2(s)}}).$$

## Proof of Theorem 2

In order to establish the asymptotic properties of $\mathrm{AUC}_t^{(j)}(\hat{\beta}_j)$ the key step to our theoretical development is the establishment of asymptotic theory for the $\hat{\beta}_j$. When there is a single cause of failure, [4] provided procedures for making inference about the maximum likelihood estimator of partial likelihood. We generalize their idea in the case of cause-specific partial likelihood estimator.

We assume the following regularity conditions hold for $i = 1, 2, .., n$

1. $\Pr(N_i^{(j)}(\tau) > 0) > 0$, where $N_i^{(j)}(\tau)$ counts number of events due to $j$-th cause of failure occurring over $[0, \tau]$.

2. Positive-definiteness of the matrices, $\Sigma_{j1}$ and $\Sigma_{j2}$ where

$$\Sigma_{j1} = \mathrm{E}\{-\frac{d}{d\beta_j} f_{ik}(\beta_j); \beta_{0j}\},$$

$$\Sigma_{j2} = 4\ \mathrm{Cov}\{g_{ik}(\beta_j), g_{ik'}(\beta_j); \beta_{0j}\},$$

where, $g_{ik}(\beta_j) = \dfrac{(f_{ik}(\beta_j)+f_{ki}(\beta_j))}{2}$ and

$$f_{ik}(\beta_j) = \int_0^\tau \int_0^\tau \mathbf{1}\{t > s\}\{\mathbf{1}\{M_i > M_k\}\frac{\frac{d}{d\beta_j}\mathrm{AUC}_{Z_i}^{(j)}(\beta_j)}{\mathrm{AUC}_{Z_i}^{(j)}(\beta_j)} - \mathbf{1}\{M_i \leqslant M_k\}\frac{\frac{d}{d\beta_j}\mathrm{AUC}_{Z_i}^{(j)}(\beta_j)}{1-\mathrm{AUC}_{Z_i}^{(j)}(\beta_j)}\} dN_i^{(j)}(s) dN_k^{(j)}(t),$$

.

3. Assume that $0 \leq \mathrm{AUC}_t^{(j)}(\beta_j) \leq 1$ and it is twice differentiable with respect to $\beta_j$. Also, we assume that $\dfrac{\delta^2\,\mathrm{AUC}_t^{(j)}(\beta_j)}{\delta\beta_j\delta{\beta_j}^T}$ negative-definite for any $t \,\epsilon\, (0, \tau)$ in a neighborhood of $\beta_{0j}$.

In practice, condition (1) can be enforced simply by not choosing $\tau$ to be greater than the maximum observation time. Condition (2) can be interpreted that the sample covariance among the covariates is asymptotically non-singular. For simplicity we assume $\beta_j$ is one-dimensional and its corresponding true value is $\beta_{0j}$. Multi-dimensional cases can be treated in a similar way.

To begin with, we prove the consistency of $\hat{\beta}_j$. Consider the Taylor expansion of $j$-th cause specific partial likelihood $L(\beta_j)$ around $\beta_{0j}$

$$\frac{1}{n^2}\{L(\hat{\beta}_j) - L(\beta_{0j})\} = \frac{1}{n^2}\frac{\delta\, L(\beta_{0j})}{\delta\, \beta_j}\hat{\beta}_j + \frac{1}{2}\frac{1}{n^2}\frac{\delta^2\, L(\beta_{0j})}{\delta\, \beta_j^2}\hat{\beta}_j^2 + O_p(1)|\hat{\beta}_j|^3.$$

Note that as $n \to \infty$, because the first order derivative of $\text{AUC}_t^{(j)}(\beta_j)$ is bounded, therefore, $\frac{1}{n^2}\frac{\delta\, L(\beta_{0j})}{\delta\, \beta_j} \to 0$. Furthermore, because of assumption (3), $\frac{1}{n^2}\frac{\delta^2\, L(\beta_{0j})}{\delta\, \beta_j^2} < 0$. As a result, $\frac{1}{n^2}\{L(\hat{\beta}_j) - L(\beta_{0j})\} < 0$, with probability going to 1. The consistency result follows. To prove asymptotic normality of $\hat{\beta}_j$. we restate the score equation $U(\beta_j)$ with respect to an underlying counting process $\{N_i^{(j)}(\tau),\, \tau > 0.\}$ which counts number of events due to $j$-th cause of failure occurring over $[0,\tau]$ and it is

$$\begin{aligned}
U(\beta_j) = \sum_{i=1}^{n}\sum_{k=1}^{n}\int_0^\tau\int_0^\tau \mathbf{1}\{t > s\}\{\mathbf{1}\{M_i > M_k\}\frac{\frac{d}{d\beta_j}\text{AUC}_{Z_i}^{(j)}(\beta_j)}{\text{AUC}_{Z_i}^{(j)}(\beta_j)} \\
- \mathbf{1}\{M_i \leqslant M_k\}\frac{\frac{d}{d\beta_j}\text{AUC}_{Z_i}^{(j)}(\beta_j)}{1 - \text{AUC}_{Z_i}^{(j)}(\beta_j)}\}dN_i^{(j)}(s)dN_k^{(j)}(t), \\
= \sum_{i=1}^{n}\sum_{k=1}^{n} f_{ik}(\beta_j).
\end{aligned}$$

To show the asymptotic Normality results of $\hat{\beta}_j$, we apply the Taylor expansion of $U(\beta_j)$ at $\beta_{0j}$

$$\sum_{i=1}^{n}\sum_{k=1}^{n} f_{ik}(\hat{\beta}_j) = 0,$$

$$\text{or, } \sum_{i=1}^{n}\sum_{k=1}^{n} f_{ik}(\beta_{0j}) + \sum_{i=1}^{n}\sum_{k=1}^{n}(\hat{\beta}_j - \beta_{0j})^T\frac{d\, f_{ik}(\beta_{0j})}{d\beta_j} + O_p(n^{-3/2}) = 0,$$

$$\text{or, } \sqrt{n}\ (\hat{\beta}_j - \beta_{0j}) = \sqrt{n}\ [-\sum_{i=1}^{n}\sum_{k=1}^{n}\frac{d\, f_{ik}(\beta_{0j})}{d\beta_j}]^{-1}\ [\sum_{i=1}^{n}\sum_{k=1}^{n} f_{ik}(\beta_{0j})] + O_p(n^{-1/2}),$$

$$\text{or, } \sqrt{n}\ (\hat{\beta}_j - \beta_{0j}) = A_{nj}^{-1}\ \sqrt{n}\ B_{nj}$$

where

$$A_{nj} = \frac{-1}{n^2}\sum_{i=1}^{n}\sum_{k=1}^{n}\int_0^{\tau}\int_0^{\tau}\mathbf{1}\{t>s\}\{\mathbf{1}\{M_i>M_k\}\frac{d}{d\beta_j}[\frac{\frac{d}{d\beta_j}\text{AUC}_{Z_i}^{(j)}(\beta_{0j})}{\text{AUC}_{Z_i}^{(j)}(\beta_{0j})}] - \mathbf{1}\{M_i \leqslant M_k\}\frac{d}{d\beta_j}[\frac{\frac{d}{d\beta_j}\text{AUC}_{Z_i}^{(j)}(\beta_{0j})}{1-\text{AUC}_{Z_i}^{(j)}(\beta_{0j})}]\}dN_i^{(j)}(s)dN_k^{(j)}(t),$$

and as $n\to\infty$, $A_{nj}\xrightarrow{P}\Sigma_{1j}$. Also,

$$\begin{aligned}
B_{nj} &= \frac{1}{n^2}\sum_{i=1}^{n}\sum_{k=1}^{n} f_{ik}(\beta_{0j})\\
&= \frac{1}{n^2}\sum_{i=1}^{n}[\sum_{k\neq i}^{n} f_{ik}(\beta_{0j}) + f_{ii}(\beta_{0j})]\\
&= \frac{1}{n^2}\sum_{i=1}^{n}[\sum_{k\neq i}^{n} f_{ik}(\beta_{0j})] \text{ since } f_{ii}(\beta_{0j})=0,\\
&= \frac{1}{n^2}\sum_{i=1}^{n}[\sum_{k<i}^{n} \frac{(f_{ik}(\beta_{0j})+f_{ki}(\beta_{0j}))}{2}],\\
&= \binom{n}{2}\sum_{i=1}^{n}[\sum_{k<i}^{n} \frac{(f_{ik}(\beta_{0j})+f_{ki}(\beta_{0j}))}{2}].
\end{aligned}$$

Here, $B_{nj}$ is a U-statistic of degree 2. Therefore, the asymptotic Normality follows by the projection theory in [5].

## Proof of Corollary 1

Since $\hat{\beta}_j$ is consistent for $\beta_{0j}$ and $\text{AUC}_t^{(j)}(.)$ is a continuous function, therefore, $\text{AUC}_t^{(j)}(\hat{\beta}_j)$ is a consistent estimator of $\text{AUC}_t^{(j)}(\beta_{0j})$. In order to prove the Normality of $\text{AUC}_t^{(j)}(\hat{\beta}_j)$, following the proof of theorem 2, we can write the

$$\hat{\beta}_j = \beta_{0j} + \Sigma_{1j}^{-1}\frac{1}{n^2}\sum_{i=1}^{n}\sum_{k=1}^{n} g_{ik}(\beta_{0j}) + O_p(n^{-1}).$$

Now, consider the Taylor expansion of $\text{AUC}_t^{(j)}(\hat{\beta}_j)$ around $\text{AUC}_t^{(j)}(\beta_{0j})$

$$\begin{aligned}
\text{AUC}_t^{(j)}(\hat{\beta}_j) &= \text{AUC}_t^{(j)}(\beta_{0j}) + \frac{d\text{AUC}_t^{(j)}(\beta_{0j})}{d\,\beta_j}(\hat{\beta}_j-\beta_{0j}) + O_p(n^{-1}),\\
&= \text{AUC}_t^{(j)}(\beta_{0j}) + \frac{1}{n^2}\sum_{i=1}^{n}\sum_{k=1}^{n}\Sigma_{j1}^{-1}g_{ik}(\beta_{0j})\frac{d\text{AUC}_t^{(j)}(\beta_{0j})}{d\,\beta_j} + O_p(n^{-1}).
\end{aligned}$$

Note that $\sum_{i=1}^{n}\sum_{k=1}^{n} \Sigma_{j1}^{-1} g_{ik}(\beta_{0j}) \dfrac{d\mathrm{AUC}_t^{(j)}(\beta_{0j})}{d\,\beta_j}$ is a U-statistic, which implies the asymptotic Normality result by the projection theorem. Again, the covariance matrix of $\hat{\beta}_j$ is $\hat{\Sigma}_{j1}^{-1}\hat{\Sigma}_{j2}\hat{\Sigma}_{j1}^{-1}$. The $\mathrm{AUC}_t^{(j)}$ after transformation with link function $\eta$ can be rewritten as

$$\eta(\mathrm{AUC}_t^{(j)}(\beta_j)) = \beta_{j0} + \sum_{l=1}^{7} \beta_{jl} t^{(p_l)} = \mathbf{A}\,{\beta_j}^T$$

where $\mathbf{A} = (1, t^{p_1}, t^{p_2}, \ldots, t^{p_7})$, and $\beta_j = (\beta_{j0}, \beta_{j1}, \ldots, \beta_{j7})$. Using the delta method we can obtain the variance of $\mathrm{AUC}_t^{(j)}(\hat{\beta}_j)$ as $[\dfrac{d}{d\beta_j}\eta^{-1}(\mathbf{A}\,{\beta_j}^T)]^2_{\beta_j=\hat{\beta}_j}\ \mathbf{A}\hat{\Sigma}_{j1}^{-1}\hat{\Sigma}_{j2}\hat{\Sigma}_{j1}^{-1}\mathbf{A}^T$.

# References


[1] Lee, A.J.: U-statistics: Theory and Practice. Routledge, 6000 Broken Sound Parkway NW (2019)

[2] Saha-Chaudhuri, P., Heagerty, P.: Non-parametric estimation of a time-dependent predictive accuracy curve. Biostatistics **14**(1), 42–59 (2013)

[3] Pepe, M.S.: The Statistical Evaluation of Medical Tests for Classification and Prediction. Medicine, Oxford (2003)

[4] Shen, W., Ning, J., Yuan, Y.: A direct method to evaluate the time-dependent predictive accuracy for biomarkers. Biometrics **71**(2), 439–449 (2015)

[5] Lehmann, E.L., D'Abrera, H.J.: Nonparametrics: Statistical Methods Based on Ranks. Holden-Day, USA (1975)
