# Supplementary material for: Inference about time-dependent prognostic accuracy measures in the presence of competing risks
Source: BMC Med Res Methodol. 2020 Aug 28;20:219. doi: 10.1186/s12874-020-01100-0 (PMC7456384; doi:10.1186/s12874-020-01100-0)
Supplement: Supplementary file 3 — this file includes description files for using r code provided in Additional file 2 for estimating cause-specific aUC(t) using cWMR and cFPL estimators. [file 12874_2020_1100_MOESM3_ESM.pdf]

Additional File 3: Description file for using R code for estimating cause-specific AUC(t) using cFPL and cWMR estimators

We describe the core R functions provided in Additional File 2 for estimating time-dependent incident/dynamic cause-specific area under the receiver operating characteristic (ROC) curve AUC(t) based on cFPL and cWMR estimators below.

1 cWMR function

The cWMR function creates estimated cause-specific AUC(t) based on a survival time and marker values as described in the section 2.3 of the manuscript.

Usage

cWMR(stime, status, marker,cause.of.interest,TargetTimes,bandwidth,alpha=.05,plot=TRUE, type="l", xlab="Time", ylab="Cause-specific AUC",main="Method: cWMR")

Arguments

1. stime: observed time = minimum(event time, censoring time) is a numeric vector
2. status: censoring indicator which can take value 0,1,2,...,J. Here, 0 means censoring and j indicates cause j ($j = 1, 2, ...,$ J)
3. marker: marker is a numeric vector
4. cause.of.interest: a number from status. The cause for which you would like to estimate incident/dynamic cause-specific AUC(t) using cWMR
5. TargetTimes: the time points t of interest
6. bandwidth: bandwidth value
7. alpha: the confidence level, default is 0.05
8. plot: TRUE or FALSE, default is TRUE

9. type: default is “l”, can be either of “p” for points, “l” for line, “b” for both

10. xlab: label for x-axis

11. ylab: label for y-axis

12. main: the title of the plot

Details

This function returns the estimated cause-specific AUC(t) value with corresponding standard error at TargetTimes t for the cWMR method. If plot=“TRUE” then the AUC(t) curve is plotted with horizontal line to indicate the null AUC value of 0.5.

Values

Returns a list of the following items:

- TargetTimes: The time points t of interest at which cause-specific AUC(t) have been computed
- Estimate: Estimated cause-specific AUC(t) at TargetTimes t for cause of interest
- SE: Estimated standard error of cause-specific AUC(t), the lower and upper limits of confidence interval with confidence level alpha.

2 cFPL function

The cFPL function creates estimated incident/dynamic cause-specific AUC(t) based on a survival data and marker values as described in the section 2.4 of the manuscript.

Usage

cFPL(stime, status, marker,cause.of.interest,TargetTimes,alpha=.05,plot=TRUE, type=“l”, xlab=“Time”, ylab=“Cause-specific AUC”,main=“Method: cFPL”)

Arguments

1. stime: observed time = minimum(event time, censoring time) is a numeric vector

2. status: censoring indicator which can take value 0,1,2,...,J. Here, 0 means censoring and j indicates cause j ($j = 1, 2, ..., $J)

3. marker: marker is a numeric vector

4. cause.of.interest: the cause for which you would like to estimate incident/dynamic cause-specific AUC(t) using cFPL.

5. TargetTimes: the time points t of interest

6. alpha: the confidence level, default is 0.05

7. plot: TRUE or FALSE, default is TRUE

8. type: default is "l", can be either of "p" for points, "l" for line, "b" for both

9. xlab: label for x-axis

10. ylab: label for y-axis

11. main: the title of the plot

Details

This function returns the estimated incident/dynamic cause-specific AUC(t) value with corresponding standard error at TargetTimes t for the cFPL method. If plot="TRUE" then the AUC(t) curve is plotted with horizontal line to indicate the null AUC value of 0.5.

Values

Returns a list of the following items:

- TargetTimes: The time points t of interest at which cause-specific area under the ROC curve AUC(t) have been computed

- Estimate: Estimated time-dependent cause-specific AUC(t) at TargetTimes t for cause of interest

- SE: Estimated standard error of cause-specific AUC(t), the lower and upper limits of confidence interval with confidence level alpha.
